# Anatomical Templates of the Midbrain Ventral Tegmental Area and Substantia Nigra for Asian Populations

**DOI:** 10.3389/fpsyt.2018.00383

**Published:** 2018-08-28

**Authors:** Yuko Nakamura, Naohiro Okada, Akira Kunimatsu, Kiyoto Kasai, Shinsuke Koike

**Affiliations:** ^1^Center for Integrative Science of Human Behavior, Graduate School of Arts and Sciences, University of Tokyo, Tokyo, Japan; ^2^Department of Neuropsychiatry, Graduate School of Medicine, University of Tokyo, Tokyo, Japan; ^3^World Premier International Research Initiative (WPI), International Research Center for Neurointelligence (IRCN), University of Tokyo Institutes for Advanced Study (UTIAS), University of Tokyo, Tokyo, Japan; ^4^Department of Radiology, IMSUT Hospital, Institute of Medical Science, University of Tokyo, Tokyo, Japan; ^5^University of Tokyo Institute for Diversity & Adaptation of Human Mind (UTIDAHM), Tokyo, Japan; ^6^Center for Evolutionary Cognitive Science at the University of Tokyo, Tokyo, Japan

**Keywords:** the ventral tegmental area, the substantia nigra, resting-state functional magnetic resonance imaging, neuromelanin-sensitive magnetic resonance imaging, the midbrain neural networks

## Abstract

Increasing evidence shows that the midbrain dopaminergic system is involved in various functions. However, details of the role of the midbrain dopaminergic system in these functions are still to be determined in humans. Considering that the ventral tegmental area (VTA) and substantia nigra (SN) in the midbrain are the primary dopamine producers, creating reliable anatomical templates of the VTA and SN through neuroimaging studies would be useful for achieving a detailed understanding of this dopaminergic system. Although VTA and SN anatomical templates have been created, no specific templates exist for the Asian population. Thus, we conducted anatomical and resting-state functional magnetic resonance imaging (rs-fMRI) studies to create VTA and SN templates for the Asian population. First, a neuromelanin-sensitive MRI technique was used to visualize the VTA and SN, and then individual hand-drawn VTA and SN regions of interests (ROIs) were traced on a small sample of neuromelanin-sensitive MRIs (dataset 1). Second, individual hand-drawn VTA and SN ROIs were normalized to create normalized VTA and SN templates for the Asian population. Third, a seed-based functional connectivity analysis was performed on rs-fMRI data using hand-drawn ROIs to calculate neural networks of VTA and SN in dataset 1. Fourth, a seed-based functional connectivity analysis was performed using VTA and SN seeds that were created based on normalized templates from dataset 1. Subsequently, a seed-based functional connectivity analysis was performed using VTA and SN seeds in another, larger sample (dataset 2) to assess whether neural networks of VTA or SN seeds from dataset 1 would be replicated in dataset 2. The Asian VTA template was smaller and located in a more posterior and inferior part of the midbrain compared to the published VTA template, while the Asian SN template, relative to the published SN template, did not differ in size but was located in the more inferior part of the midbrain. The neural networks of the VTA and SN seeds in dataset 1 were replicated in dataset 2. Altogether, our normalized template of the VTA and SN could be used for measuring fMRI activities related to the VTA and SN in the Asian population.

## Introduction

Increasing evidence suggests that the midbrain dopaminergic system is involved in various behavioral and cognitive functions, such as motor behavior, working memory, decision making, attention, reward-related learning, addiction, and motivation (1–3). However, details of the role of the midbrain dopaminergic system in those functions remain to be determined in humans.

Recent remarkable developments of neuroimaging techniques provide the means by which to develop deeper understanding of this dopaminergic system in humans. In particular, considering that the ventral tegmental area (VTA) and substantia nigra (SN) in the midbrain are the primary dopamine producers, localizing these regions in humans is necessary if future neuroimaging studies are to reveal the role of the midbrain dopamine system.

The SN, which lies dorsal to the cerebral peduncles, is the largest nucleus in the midbrain. Dopamine neurons in the SN project to the dorsal striatum via the nigostriatal pathway. These neurons are critical for controlling voluntary movement and are associated with prediction error and processing saliency of environmental stimuli (4). Loss of dopamine neurons in the SN is an established cause of Parkinson's disease, which is characterized by tremor, rigidity, and slowness of movements (5).

The VTA is a small region in the midbrain that is contiguous to the SN. The VTA is close to the midline on the floor of the midbrain. Dopamine neurons in the VTA project to the nucleus accumbens and other various brain regions, including the amygdala, hippocampus, ventral pallidum, periaqueductal gray matter, bed nucleus of the stria terminalis, olfactory tubercle, locus coeruleus, and medial prefrontal cortex (mPFC). VTA dopamine neurons play a central role in reward-related and goal-directed behaviors (6). fMRI and PET studies have indicated that neural activity in the VTA dopamine region is associated with addiction and drug-seeking behaviors (7).

Human neuroimaging studies require reliable anatomical templates of the VTA and SN to examine their neural functions. However, creating reliable VTA and SN templates has been challenging until recent developments in MRI techniques because the VTA is contiguous with the SN and located within an approximately 20-mm cubic region in the midbrain, and it is difficult clearly detect these regions with T1- and T2-weighted images.

Murty et al. created anatomical templates of the VTA and SN regions in the midbrain using a conventional T1-weighted MRI [T1-weighted fast spoiled gradient echo (FSPGR) sequence MR image] and hand-drawn individual VTA and SN regions of interests (ROIs) (8). However, VTA or SN masks for the Asian populations have not been created. A morphometric MRI study suggested that the Caucasian and Asian brains differ in shape, size, and multiple local structures (9–11); briefly, there exist ethnicity-based structural brain differences between the Caucasian and Asian brains. Thus, we designed the current study to create the Asian anatomical templates of the VTA and SN to facilitate neuroimaging studies in Asian populations.

Previous findings have suggested that the SN and VTA functional networks are different (8, 12) therefore, we speculated that appropriate VTA and SN templates could elucidate significantly different neural networks. To test this hypothesis, VTA, and SN functional networks were created by applying a seed-based functional connectivity analysis to rs-fMRI data, using Asian VTA and SN seeds.

## Materials and methods

### Participants

We collected two datasets: dataset 1 consisted of 16 healthy participants (11 males, 5 females; age mean = 21.3 years, *SD* = 8.1, range = 13–43 years) and dataset 2 of 61 healthy participants (25 males, 36 females; age mean = 39.4 years, *SD* = 8.1, range = 27–59 years). All participants provided written informed consent, and the study was approved by the ethics committee at the Department of Medicine, the University of Tokyo (No. 3150-20).

We have also downloaded the NKI-Rockland FSPGR data (13) from fcon 1,000 project website, along with demographic assessments (http://fcon_1000.projects.nitrc.org/indi/pro/nki.html) and selected 75 Caucasian samples matched in age and gender (42 male, 33 female, age mean = 43.5, *SD* = 19.6, range 18–85) to examine the morphological differences between the Caucasian and Asian brains in the midbrain area.

### Image acquisition

All images from participants in dataset 1 were collected using a MAGNETOM Prisma 3.0 Tesla scanner equipped with a 64-channel head coil (Siemens Healthineers, Erlangen, Germany). All images from participants in dataset 2 were collected using a Discovery MR750w 3.0 Tesla scanner equipped with a 24-channel head coil (GE Healthcare, Chicago, Illinois, United States).

We applied a newly developed neuroimaging technique, namely neuromelanin-sensitive magnetic resonance imaging, to create individual hand-drawn VTA and SN templates for dataset 1. This imaging technique can visualize tissues containing neuromelanin, such as the SN, and has been utilized in neuroimaging studies to examine the SN and VTA (14–17). The neuromelanin-sensitive MRI used the following pulse sequence: T1-weighted fast spin echo (TR/TE = 24.0/2.20 ms, field of view = 220 mm^2^, matrix = 320 × 320, voxel size = 0.3 × 0.3 × 2.5 mm^3^) with an acquisition time of 7 min 12s.

Blood oxygen level-dependent functional MR imaging (BOLD fMRI) data for dataset 1 were acquired using the following pulse sequence: single-shot echo-planar sequence (TR/TE = 2,500/30 ms, flip angle = 80°, field of view = 212 mm^2^, matrix = 64 × 64, voxel size = 3.3 × 3.3 × 4.0 mm^3^) with an acquisition time of 10 min 17s. Thirty-eight contiguous slices were acquired. Whole-brain high-resolution anatomical image data were obtained using the following pulse sequence: T1-weighted 3D MPRAGE (TR/TE = 1,900/2.53 ms, flip angle = 9°, field of view = 212 mm^2^, matrix = 256 × 256, voxel size = 1.0 × 1.0 × 1.0 mm^3^) with an acquisition time of 4 min 26s. For dataset 2, BOLD fMRI data were acquired using the following pulse sequence: single-shot echo-planar sequence (TR/TE = 2,500/30 ms, flip angle = 80°, field of view = 212 mm^2^, matrix = 64 × 64, voxel size = 3.3 × 3.3 × 4.0 mm^3^) with an acquisition time of 10 min 17 sec. Forty contiguous slices were acquired. Whole-brain high-resolution anatomical image data were obtained using the following pulse sequence: T1-weighted 3D FSPGR (TR/TE = 7.7/3.1 ms, flip angle = 11°, field of view = 240 mm^2^, matrix = 256 × 256, voxel size = 1.0 × 1.0 × 1.2 mm^3^), with acquisition time of 4 min 26s.

### Creating VTA and SN ROIs

#### Criteria for creating hand-drawn VTA and SN ROIs

**Figure 1:**
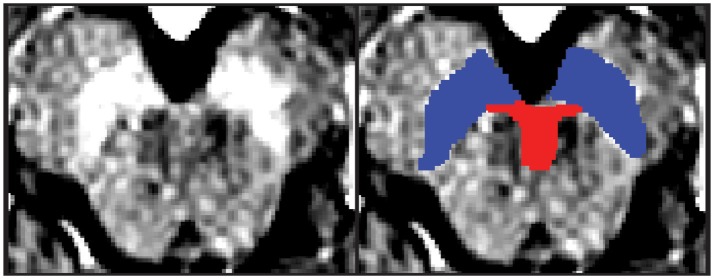
One example of the midbrain axial slice and hand-drawn ROIs. The left panel depicts the midbrain axial slice of one participant. The right panel depicts hand-drawn VTA and SN ROIs. Red, Ventral tegmental area; Blue = Substantia nigra.

Regions of interest (ROIs) were hand-drawn on neuromelanin-sensitive MR images of individual participants in dataset 1 using FSLView, which is a viewer function of FSL (18). First, the SN was defined as a high intensity area in the midbrain. Then, the VTA was defined along the SN. The ventral margin of the VTA was defined along the margin of the midbrain. The lateral margin of the VTA was defined as the medial margin of the SN. The rostral margin of the VTA was defined as lying at the same level as the red nucleus, which was depicted as a low intensity area (Figure 1). Two authors (YN and SK) independently defined the VTA and SN for dataset 1, and the obtained volumes had substantial reliability within the two raters (SN: ICC(2,k) = 0.68, VTA: 0.73).

#### Asian template of the midbrain ROIs

To create normalized templates for the Asian populations, each participant's neuromelanin-sensitive MR image was linearly transformed into an anatomical image space and a transfer matrix (matrix i) was created using FMRIB's linear image registration tool (FLIRT), which is implemented in FSL (19). Subsequently, the participant's anatomical image was linearly transformed into the standard space and a matrix for this transfer (matrix ii) was created using FLIRT. These two matrixes (matrix i and matrix ii) were then concatenated to create a matrix for a linear transfer from a neuromelanin-sensitive MR image space to the standard space (matrix iii). Each participant's hand-drawn ROIs were transferred to the standard space using matrix iii with FLIRT. Then, all normalized individual ROIs were binarized, consisting of a value of 1 for regions within each ROI and 0 for regions outside the ROI, and averaged across all participants using fslmaths tool as implemented in FSL. Averaged VTA and SN ROIs were considered as normalized probabilistic templates for VTA and SN, respectively.

### Voxel-based morphometry analysis for the midbrain region in Asian and Caucasian populations

We performed voxel-based morphometry (VBM) analysis to examine the morphological differences between Caucasian and Asian brains. T1-weighted images in Caucasian and Asian samples from dataset 2 were processed with FMRIB Software Library VBM (FSL-VBM) (18), using VBM (20, 21). First, skull, dura, and other nonbrain tissues were removed with the brain extraction tool (22). Next, tissue type segmentation was preformed using FMRIB's Automated Segmentation Tool (FAST4) (23). The resulting gray matter partial volume images were then aligned to MNI152 standard space using the affine registration tool FLIRT, followed optionally by nonlinear registration using FMRIB's nonlinear image registration tool (FNIRT), which uses a b-spline representation of the registration warp field (24). The resulting images were averaged to create a study-specific template, to which the native gray matter images were then nonlinearly reregistered. To correct for local expansion or contraction, we modulated the registered partial-volume images by division with the Jacobian of the warp field. The modulated segmented images were then smoothed with an isotropic Gaussian kernel with a sigma of 2 mm. To compare gray matter volumes in the midbrain between Asian and Caucasian samples, we performed an independent t-test. A voxelwise statistical map was computed using permutation testing, using a false-positive cutoff of 5% corrected for multiple comparisons. To examine details of the differences of midbrain volumes in the midbrain region, we applied a midbrain anatomical template from Harvard-Oxford subcortical structural atlas (25, 26) to a voxelwise statistical map.

### Preprocessing rs-fMRI data

Conventional preprocessing was performed for dataset 1 and dataset 2 using tools from the FMRIB Software Library (FSL Version 5.0; http://www.fmrib.ox.ac.uk/fsl/) package (18, 27, 28). fMRI data were preprocessed as follows: (1) head motion correction by realigning the time series to the middle volume (29), (2) removing non-brain material using the brain extraction tool (22), (3) slice-timing correction using Fourier-space phase shifting, aligning to the middle slice (30), (4) image smoothing with a 5-mm full-width at half-maximum Gaussian kernel, (5) high-pass filtering with a 150 s cutoff, (6) low-pass filtering with a 1 s cutoff and (7) grand-mean intensity normalization using a single multiplicative factor. To remove the effects of time-points that were corrupted by large motion (motion outliers) from the analysis, a confound matrix was created to be used at the participant level analysis using FSL's toolbox (31). Head motion, which lied outside 1.5 times the interquartile range above the upper quartile of head motion across the whole scan, was defined as a motion outlier. This confounder matrix also included a time series extracted from individual white matter and cerebrospinal fluid regions. Before group analyses, the high-resolution anatomical image was normalized to the MNI avg152 T1-weighted template (2-mm isotropic resolution) using a nonlinear transformation with a 10-mm warp resolution, as implemented by FSL's fMRI non-linear registration tool.

### Seed-based functional connectivity analysis

#### Functional connectivity maps of hand-drawn ROIs

For dataset 1, connectivity maps of each ROI were created using hand-drawn VTA and SN ROIs. First, a matrix (matrix 1) to transfer a neuromelanin-sensitive MR image space to an anatomical image space was calculated using FLIRT. Second, a matrix (matrix 2) to transfer from an anatomical image space to a functional image space was calculated using FLIRT. Third, matrix 1, and matrix 2 were combined to transfer hand-drawn VTA and SN images from a neuromelanin-sensitive MR image space to a functional image space using FLIRT (matrix 3). Finally, hand-drawn VTA and SN regions were transferred to a functional image space from a neuromelanin-sensitive MR image space using matrix 3.

Time-series extraction from each hand-drawn ROI was performed on non-smoothed preprocessed fMRI data. To create functional connectivity maps, the extracted time-series from hand-drawn VTA and SN ROIs were included in a regression model (the general linear model; GLM) using FSL's fMRI Expert Analysis Tool (FEAT). This model also included a confounder matrix. In the participant-level analysis, four contrasts were created as follows: (1) VTA connectivity map, (2) SN connectivity map, (3) VTA-SN connectivity map, and (4) SN-VTA connectivity map. Individual contrast maps entered the group-level analysis to perform one sample *t*-tests. Clusters that survived *p* < 0.05 (a cluster-extent based threshold for a family-wise error (FWE) correction) were considered as significant.

#### Functional connectivity maps of Asian template seeds

For dataset 1 and dataset 2, connectivity maps using VTA and SN seeds were created. VTA and SN seeds were generated based on normalized probabilistic templates for VTA and SN. A 3 mm-sphere at the peak of mass for the VTA template was created as the VTA seed and a 3 mm-sphere at the bilateral peaks of mass for each SN template were created and merged as the SN seed.

To create connectivity maps using each seed, time-series extraction from each seed was performed on non-smoothed preprocessed fMRI data. The extracted time-series data and a confounder matrix entered the regression model and participant-level contrasts were created for the VTA connectivity map, SN connectivity map, VTA-SN connectivity map, and SN-VTA connectivity map. These contrasts entered the group-level analysis to perform one sample *t*-tests. Clusters that survived *p* < 0.05 (a cluster-extent based threshold for FWE correction) were considered as significant.

## Results

### Hand-drawn VTA and SN ROIs

The mean volume of the VTA and SN ROIs across all participants in dataset 1 was 357.63 ± 108.35 mm^3^ and 1087.70 ± 227.53 mm^3^ (mean ± SD), respectively. Our VTA ROI was significantly smaller than published VTA templates (*p* = 0.022), whereas our SN ROI did not differ from the published SN template (*p* = 0.132) (8). Figure 1 shows an example of the hand-drawn VTA and SN ROIs for a participant.

### Morphological differences in the templates and midbrain between Asian and Caucasian populations

#### The peak voxels of each probabilistic template

**Figure 2:**
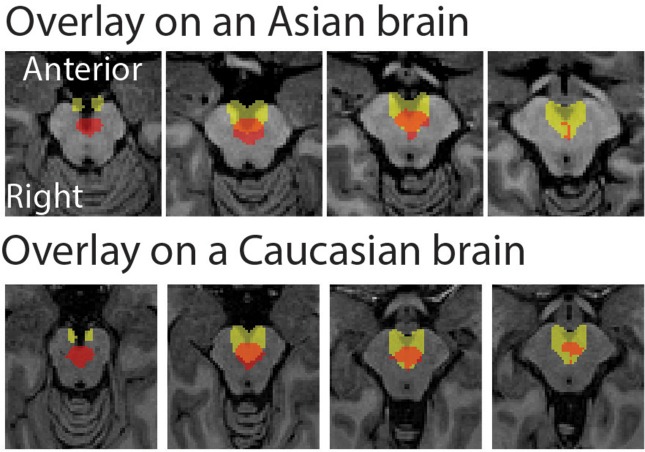
Morphological differences between Asian and published VTA templates. Asian (red) and published (yellow) templates of VTA transferred to an individual space were overlay on an Asian sample from dataset 2 (the upper raw), and that of VTA templates were overlay on a Caucasian template (the bottom row).

**Figure 3:**
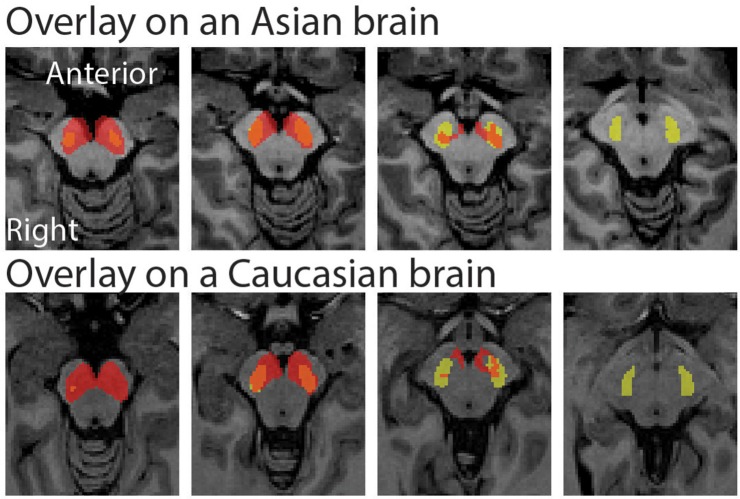
Morphological differences between Asian and published SN templates. Asian (red) and published (yellow) templates of SN transferred to an individual space were overlay on an Asian sample from dataset 2 (the upper raw), and that of SN templates were overlay on a Caucasian template (the bottom row).

The peak coordinate of the Asian VTA probabilistic template was [x, y, z] = [−2, −20, −18], while that of published VTA was [x, y, z] = [−2, −14, −18]. The peak coordinate of the Asian SN probabilistic template was [x, y, z] = [−6, −20, −18] (left side) and [8, −24 −18] (right side), whereas that of published SN was [x, y, z] = [−12, −22, −10] (left side) and [12, −20, −12] (right side). To examine the locations of probabilistic templates, we overlaid the Asian and Caucasian templates on individual Asian anatomical brain from dataset 2 and Caucasian anatomical brain respectively (Figures 2, 3). Our templates fit well with each of the Asian midbrain regions. In contrast, Caucasian templates were located more anteriorly, and the anterior part of these templates covered the fourth ventricle, compared to the Asian templates (Figures 2, 3).

#### Volumes of the midbrain

The independent *t*-test showed that gray matter volumes in the midbrain in the Caucasian samples were larger than that of the Asian samples from the dataset 2 (*p* < 0.001). The peak voxel was in the medial posterior part of the midbrain ([x, y, z] = [6, −40, −24]).

### Functional connectivity maps of hand-drawn ROIs

#### Functional connectivity map of each ROI

**Figure 4:**
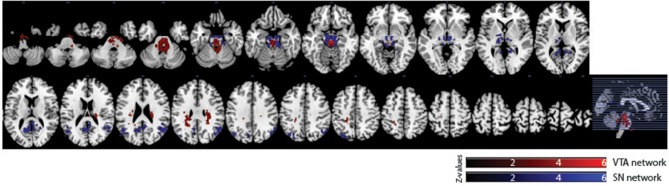
Significant VTA and SN functional connectivity maps. *p* < 0.05, whole-brain corrected. Red clusters = VTA contrast, Blue clusters = SN contrast.

Of the 16 participants in dataset 1, 13 rs-fMRI data were available for the analysis. The hand-drawn VTA connectivity map included the midbrain (VTA), while that of the SN included the midbrain (SN), thalamus, pallidum, posterior cingulate cortex (PCC), and angular gyrus (Figure 4, Table 1).

**Table 1:** Peak coordinates of regions of each connectivity map.

**Region**	**Cluster size (voxels)**	***z*-value**	**MNI coordinate**		
			**x**	**y**	**z**
**HAND-DRAWN ROIs (DATASET 1)**					
**VTA seed**
VTA	1545	5.33	−2	−26	−16
White matter	355	3.65	−20	−48	26
**SN seed**					
SN	1767	4.99	6	−28	−18
Thalamus^*^		4.60	8	−16	−4
Pallidum^*^		3.65	−18	−16	−2
PCC	1031	4.45	4	−42	14
Angular Gyrus (Right)	617	4.29	54	−54	18
Angular Gyrus (Left)	503	3.96	−54	−60	26
**ASIAN**-**VTA SEED (DATASET 1)**					
VTA	1281	6.26	2	−22	−20
Brain stem (Pons)	917	4.59	−18	−20	−20
PCC	249	4.08	6	−24	38
**ASIAN**-**VTA SEED (DATASET 2)**					
VTA	16625	14.8	−2	−24	−18
Frontal pole	6476	5.45	−16	38	54
Precuneus cortex	1769	4.90	−4	−60	18
Lateral occipital cortex	580	4.70	−48	−72	38
Cerebellum	227	4.30	36	−78	−48
Amygdala^*^		5.36	−24	−8	−24
Middle temporal gyrus^*^		5.47	−54	−12	−22
**ASIAN**-**SN SEED (DATASET 1)**					
SN	1219	5.34	10	−22	−16
Thalamus^*^		2.98	8	−12	−4
Pallidum^*^		2.97	−24	−16	−2
Occipital fusiform gyrus	374	4.11	−32	−66	−20
**ASIAN**-**SN SEED (DATASET 2)**					
SN	19229	13.0	8	−22	−18
Thalamus^*^		6.38	10	−24	−6
Pallidum^*^		5.00	−20	−12	−2
Angular gyrus	155	3.71	52	−50	58
PCC	24	2.95	−8	−36	24
Precuneus	21	2.93	−24	−52	6
Superior frontal gyrus	10	3.01	20	18	68

* *, subpeaks*.

#### Differences between connectivity maps of each ROI

We also observed distinct functional connectivity maps of the VTA and SN. Compared to the SN connectivity map, the VTA connectivity map showed greater functional connectivity in the caudate/white matter (Figure 6). Conversely, compared to the VTA connectivity map, the SN connectivity map showed greater functional connectivity in the PCC, lateral occipital cortex, thalamus, paracingulate gyrus, and inferior frontal gyrus (Figure 6).

### Functional connectivity maps of Asian template seeds

#### Functional connectivity map of each seed

**Figure 5:**
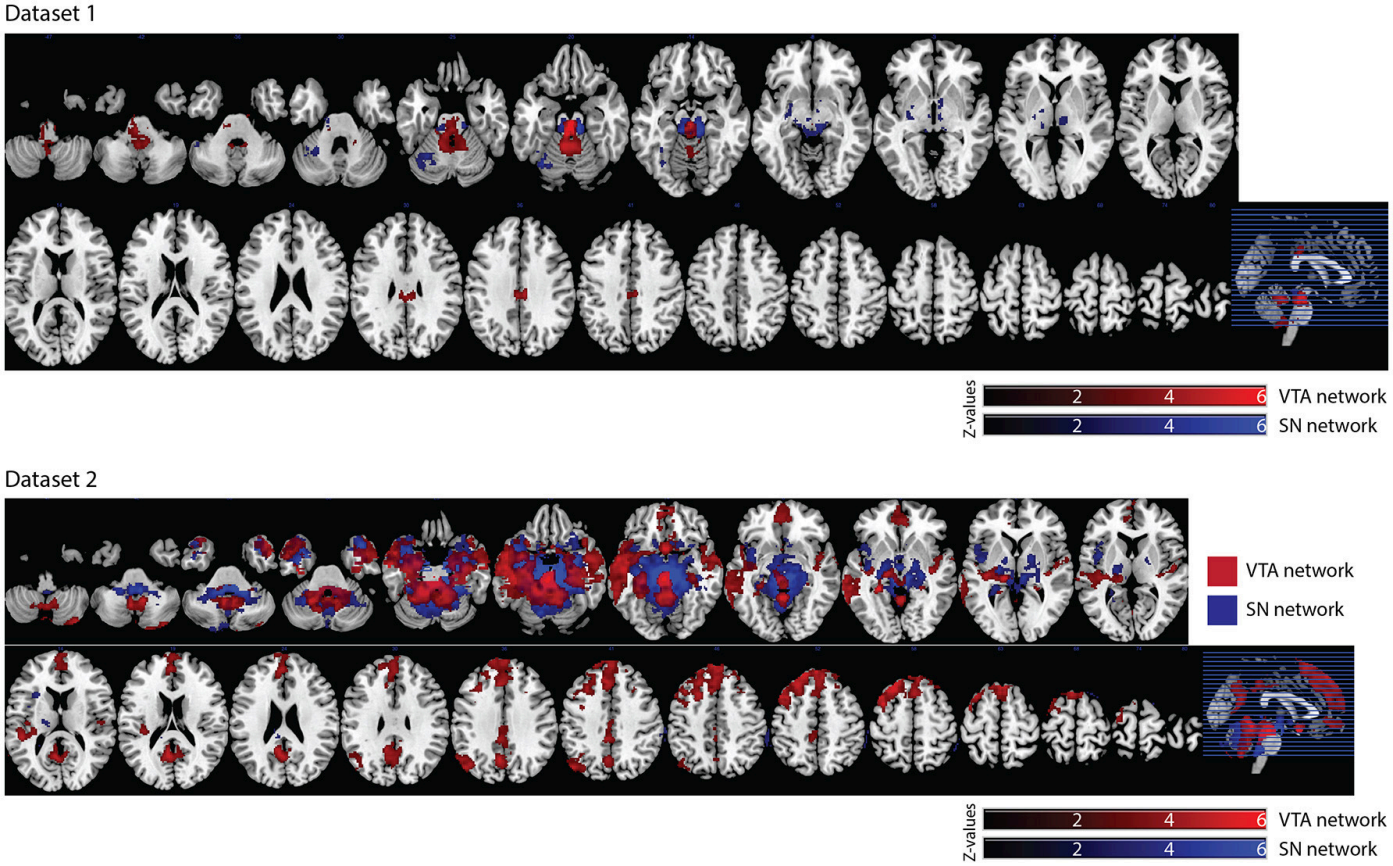
Significant VTA and SN functional connectivity maps. *p* < 0.05, whole-brain corrected. Red clusters = VTA contrast, Blue clusters = SN contrast.

In dataset 1, the connectivity map of the Asian-VTA seed included the midbrain (VTA), brain stem, and PCC, while that of the Asian-SN seed included the midbrain (SN), thalamus, pallidum, and occipital fusiform gyrus (Figure 5, Table 1). These maps were similar to the functional connectivity maps of the hand-drawn VTA and SN ROIs.

In dataset 2, the connectivity map of the Asian-VTA seed included the midbrain (VTA), frontal pole, precuneus, lateral occipital cortex, cerebellum, amygdala, and middle temporal gyrus, whereas that of the Asian-SN seed included the midbrain (SN), thalamus, pallidum, angular gyrus, PCC, precuneus, and superior frontal gyrus (Figure 5, Table 1). These maps overlapped notably with the connectivity maps derived from dataset 1.

#### Differences between connectivity maps of each seed

**Figure 6:**
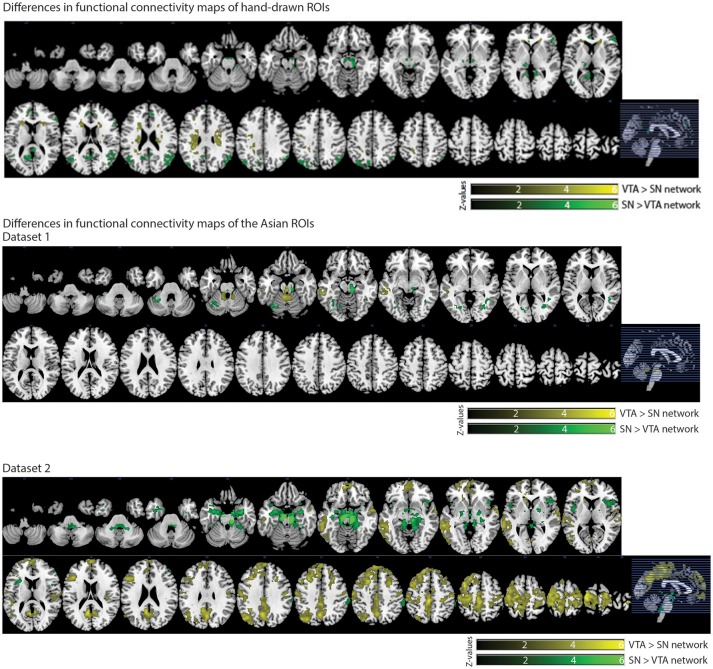
Significant differences in functional connectivity maps between VTA and SN seeds. *p* < 0.05, whole-brain corrected. Yellow clusters, VTA > SN contrast; Green clusters, SN > VTA contrast.

In dataset 1, compared with the Asian-SN connectivity map, the Asian-VTA connectivity map showed greater functional connectivity in the midbrain (VTA) and middle temporal gyrus (Figure 6), whereas the Asian-SN connectivity map showed greater functional connectivity in the midbrain (SN) and occipital fusiform gyrus, in comparison with that of the VTA (Figure 6).

In dataset 2, compared to the Asian-SN connectivity map, the Asian-VTA connectivity map showed greater functional connectivity in the precentral gyrus, superior temporal gyrus, lateral occipital cortex, and frontal pole (Figure 6). Conversely, compared with the VTA connectivity map, the SN connectivity map showed greater functional connectivity in the midbrain (SN), frontal operculum cortex, central opercular cortex, supramarginal gyrus, and frontal pole (Figure 6).

## Discussion

We created VTA and SN templates using hand-drawn ROIs for the Asian population. The Asian-VTA template was smaller and located in the more posterior part of the midbrain compared to that of the published VTA template, whereas the Asian-SN template, relative to that of the published SN, did not differ in the size, but was located more inferiorly.

As we expected, seed-based functional connectivity analysis segregated distinct functional connectivity maps for the Asian VTA and SN seeds. These maps from dataset 2 were similar to the connectivity maps of hand-drawn VTA and SN ROIs from dataset 1. Connectivity maps of the Asian seeds from dataset 2 overlapped with connectivity maps from dataset 1. In addition, connectivity maps of the Asian VTA and SN seeds shared regions with connectivity maps of published VTA and SN seeds. Considering these results, our normalized templates of the VTA and SN would be of utility as anatomical templates for neuroimaging studies in Asian populations.

### VTA and SN templates for Asian populations

Previous MRI studies have reported SN volumes, which were measured based on MRI images, of nearly 1,000 mm^3^[1,103.7 mm^3^ (32), 1,006 mm^3^ (33), and 972.38 mm^3^ (8)], which approximate our SN ROI volume (1087.70 ± 227.53 mm^3^). In contrast, VTA volumes have rarely been reported and the values are inconsistent: 1018 mm^3^ (34), 159.9 mm^3^ (35), and 440.98 mm^3^ (8), all of which are quite different from our VTA ROI volume (357.63 ± 108.35 mm^3^). These inconsistencies in VTA volumes are likely caused by morphological traits of the VTA. Since the VTA is small and is contiguous with the SN, extra caution would be required to segregate the VTA and SN. In addition, methodological differences among studies could be another cause of these inconsistencies; one study used a 7-Tesla MRI scanner (35), whereas the others used 3-Tesla MRI scanners (8, 34). Further studies are needed to determine the applicable VTA ROI using MRI.

The probabilistic Asian-VTA template was well localized within the central part of the midbrain. The most probable region of the VTA template was localized on slightly left of center of the midbrain at the same horizontal level as the red nucleus. The probabilistic Asian-SN template was contiguous with the VTA template and localized within the midbrain. The most probable region of the SN template was located at the same horizontal level as the VTA template and at a more lateral part of the midbrain. Altogether, these Asian templates represent morphological traits of the human VTA and SN (36).

### Morphological differences in the midbrain area between the Asians and Caucasians

The Asian-VTA template was smaller and located in the more posterior part of the midbrain compared to that of the published VTA template, whereas the Asian-SN template, relative to that of the published SN, did not differ in size but was located more downwards. In our samples, gray matter volume of the Asian samples was smaller than that of the Caucasian samples. Although, to the best of our knowledge, there has been no study to compare the Caucasian and Asian VTA or SN templates directly, some studies have compared the brain structures between Asians and Caucasians. A previous morphological MR image study has reported that volumes of the brain stem of Eastern Asians were greater than that of the Caucasians (9). Whereas, a morphological study has reported that the brain stem of Eastern Asian samples was smaller than that of Caucasian samples (10), and another study has showed that total intracranial volume of Eastern Asian samples was smaller than that of Western Asian samples (11). Although that previous findings about of brain morphological differences among races are not consistent, it is likely that the midbrain regions morphologically differ between ethnicities as we have observed in this study.

### Neural networks of VTA and SN templates

Functional connectivity is defined as the temporal dependence of neuronal activity patterns of anatomically separated brain regions. Functional connectivity between brain regions, as determined by the co-activation of functional MRI time-series at rest, is purported to reflect functional relationships among such regions (37). Accordingly, seed-based connectivity analysis of rs-fMRI data is likely to be adequate to segregate neural networks of the VTA and SN.

With respect to the VTA neural network, the connectivity map of the hand-drawn VTA was similar to that of the Asian-VTA seed in dataset 1. Significant overlap was observed in the midbrain. In contrast, some parts of the connectivity map of the Asian-VTA seed from dataset 2 did not overlap with that of the hand-drawn and Asian-VTA seed from dataset 1, such as the frontal pole, precuneus, and lateral occipital cortex.

The connectivity map of the hand-drawn SN from dataset 1 significantly overlapped with that of the Asian-SN seed from dataset 1 in the midbrain regions, thalamus, and pallidum. Additionally, the connectivity map of the hand-drawn SN also overlapped with that of the Asian-SN seed from dataset 2 in the midbrain region, thalamus, pallidum, and angular gyrus. However, some parts of the connectivity map from dataset 2 did not overlap with that of the hand-drawn or Asian-SN seed from dataset 1 (e.g., PCC, precuneus, and superior frontal gyrus). Using different MRI scanners could lead these discrepancies in connectivity maps for the VTA or SN. In addition, differences in sample sizes is another possible cause of these discrepancies. In addition, morphological difference between the hand-drawn ROIs and the Asian-seeds could cause minor differences in each neural network from the same dataset (dataset 1).

Our results partially replicated previous findings. Murty et al performed seed-based functional connectivity analysis on rs-fMRI data using their VTA and SN seeds. They reported that the VTA had significant connectivity with the anterior brain stem and subgenual cingulate compare to that of SN; these results were also observed in the current study (8). Tomasi and Volkow also performed seed-based rs-fMRI connectivity analysis using VTA and SN masks created based on coordinates. They reported that the VTA had significant connectivity with the amygdala and the SN was connected to the pallidum and thalamus, as we observed in the current study (38).

Unlike the results of Murty et al. (8), there was no significant difference in the connectivity maps between VTA and SN seeds in the superior parietal lobule, pre-/postcentral gyrus, superior frontal gyrus, supplementary motor cortex. These inconsistencies are likely due to different sample sizes and morphological differences in templates. To create VTA and SN templates and perform a seed-based connectivity analysis, Murty et al. recruited 50 participants, while we collected 13 samples for dataset 1 and 61 samples for dataset 2. In addition, the peak voxel coordinates of published templates are different from our data. Considering these differences in sample size and templates, we thus did not find differences between connectivity maps of VTA and SN in the regions they have reported by them.

Previous animal and human anatomical studies have reported that the VTA significantly contributes to the mesolimbic and mesocortical pathways including the ventral striatum, amygdala, and prefrontal cortex, whereas the SN contributes to the nigrostriatal pathway including the caudate, putamen, pallidum, and thalamus (12, 39–41). Consistent with previous non-human primate studies, we found that the VTA had neural connectivity with the amygdala, prefrontal cortex, while the SN was connected to the pallidum and thalamus (42). Furthermore, a human anatomical neuroimaging study using diffusion tensor imaging showed that the VTA has significant connectivity with the whole brain, including the amygdala and prefrontal cortex, and the SN is connected to the pallidum and thalamus, consistent with our results (43). Overall, the neural networks of our normalized templates included brain regions that have been reported to have anatomical neural connections with the VTA and SN.

### Limitations

The present results haveto be considered in light of the following limitations. First, the template we created would not necessarily represent the VTA and SN in the general population because of the relatively small sample size. However, we confirmed that the templates fit well with all Asian brains from dataset 2 and connectivity maps of the hand-drawn ROIs were similar to those derived from normalized seeds in the same dataset. In addition, connectivity maps of normalized seeds in dataset 1 were similar to those in dataset 2. Considering these results, our templates likely represent the VTA and SN in Asians is advisable. However, replication of these results via a study with a relatively large sample of Asians would be advised. Second, although the VTA and SN are the major source of dopamine, the neural networks we observed would not necessarily be the dopaminergic neural networks because rs-fMRI cannot reveal neural networks that depend on specific neurotransmitters. Further studies are required, such as nuclear MRI studies (44) to examine dopaminergic neural networks of the VTA and SN. Third, rs-fMRI data cannot revealed neural networks that are active during task execution. Given that an animal study showed different neural networks activations in the VTA and SN in a task-contingent manner (45), a task-based study or a study including an index of task-dependent neural activity is required to understand these task-dependent neural networks of the VTA and SN.

### Conclusion

We created VTA and SN templates for Asian population, which fit well on Asian midbrains. In addition, a seed-based functional connectivity analysis using hand-drawn VTA and SN regions as well as normalized VTA and SN seeds showed that connectivity maps for hand-drawn ROIs and normalized seeds were similar to each other in dataset 1. Furthermore, connectivity maps of normalized seeds in dataset 1 overlapped notably with connectivity maps from dataset 2. Besides, in dataset 1 and 2, a seed-based functional connectivity analysis using hand-drawn ROIs or normalized seeds showed distinct connectivity maps for each seed. Given these results, our templates of the VTA and SN likely reflect the VTA and SN in healthy Asian populations.

## Author contributions

YN analyzed data and drafted the manuscript. SK supervised data analysis and drafted the manuscript. NO, AK, and KK contributed to obtaining MRI dataset 2. All authors approved the final manuscript.

### Conflict of interest statement

The authors declare that the research was conducted in the absence of any commercial or financial relationships that could be construed as a potential conflict of interest.
